# Tool use for corpse cleaning in chimpanzees

**DOI:** 10.1038/srep44091

**Published:** 2017-03-13

**Authors:** Edwin J. C. van Leeuwen, Katherine A. Cronin, Daniel B. M. Haun

**Affiliations:** 1 University of St Andrews, School of Psychology & Neuroscience, Westburn Lane, St Andrews, Fife, KY16 9JP, United Kingdom; 2 Max Planck Institute for Psycholinguistics, Wundtlaan 1, 6525 XD Nijmegen, The Netherlands; 3 Lincoln Park Zoo, Lester E. Fisher Center for the Study and Conservation of Apes, IL 60614, Chicago, United States of America; 4 University of Leipzig, Department of Early Child Development and Culture and Leipzig Research Center for Early Child Development, Jahnallee 59, Leipzig, 04109, Germany

## Abstract

For the first time, chimpanzees have been observed using tools to clean the corpse of a deceased group member. A female chimpanzee sat down at the dead body of a young male, selected a firm stem of grass, and started to intently remove debris from his teeth. This report contributes novel behaviour to the chimpanzee’s ethogram, and highlights how crucial information for reconstructing the evolutionary origins of human mortuary practices may be missed by refraining from developing adequate observation techniques to capture non-human animals’ death responses.

## Results

Social tool use is virtually absent in the animal kingdom. Apart from an early report by McGrew & Tutin^1^,^2^, all accounts of non-human animal tool use are in the context of individual activities (e.g. refs 3, 4, 5). Here, we report a new case of social tool use in chimpanzees in a very unusual context: grass tool use for dental corpse cleaning.

Noel, a wild-born female chimpanzee living at the Chimfunshi Wildlife Orphanage Trust in Zambia, attended the dead body of Thomas, a nine-year-old male whom she adopted (*sensu*^6^) when his mother died four years earlier. At the time of the reported observations, Noel was 33 years old. She was brought to Chimfunshi when she was 14 years old, after spending most of her life at a private home in former Zaïre. Autopsy revealed that Thomas had most likely died from a combination of a viral and bacterial lung infection (for more details, see ref. 7). The majority of the group visited Thomas’ body at least once^7^, but when most chimpanzees were lured away with highly attractive food, Noel remained at his body and cleaned his teeth with a grass tool. Nina, her adolescent daughter, stayed at her side and observed the cleaning efforts of her mother. In more detail, this unique event consisted of the following actions.

**Figure 1:**
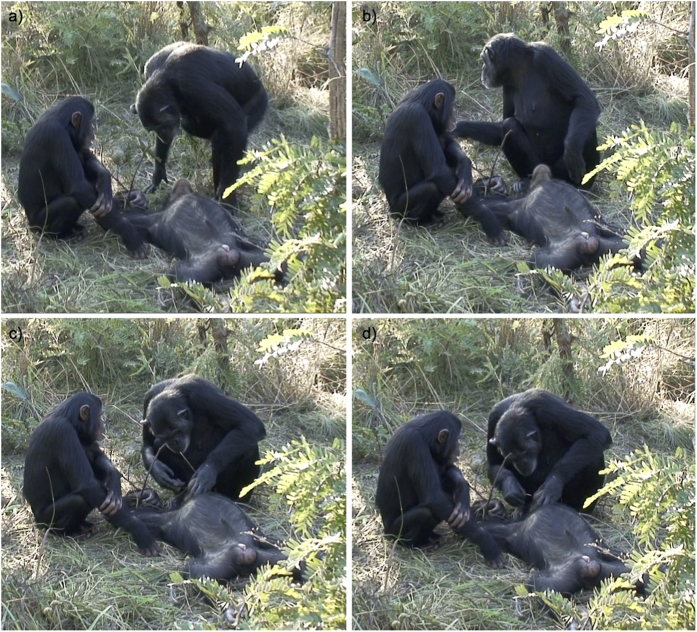
Tool use for corpse cleaning in chimpanzees. A female chimpanzee named Noel (**a**) approached Thomas’ body, (**b**) turned sideways to select a hard piece of grass, (**c**) held the grass in her mouth while opening Thomas’ mouth with both of her hands, and (**d**) cleaned Thomas’ teeth using the grass.

**Figure 2:**
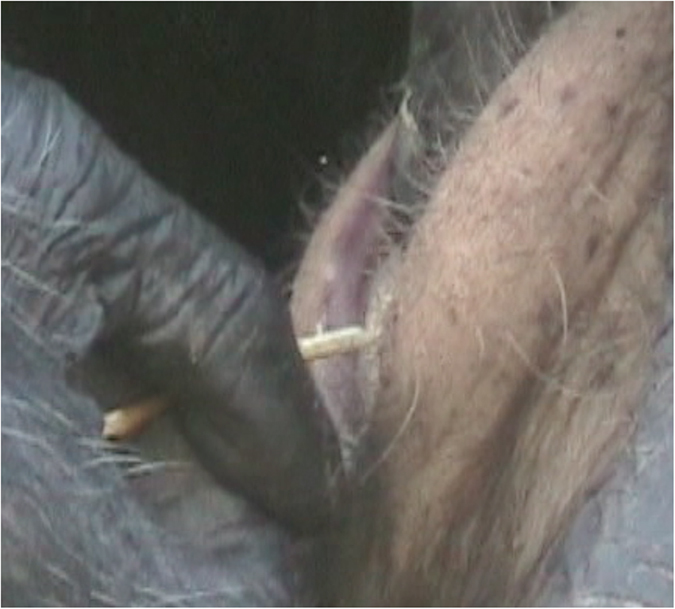
Hand-tool dexterity. Depicted is the way by which Noel held her hands and the grass tool in relation to Thomas’ mouth while cleaning his teeth.

Noel approached Thomas’ body, sat down close to his head, turned her upper body sideways to select a hard piece of grass, put the grass in her mouth, and opened Thomas’ mouth with both of her hands. Then she wrapped her fingers around Thomas’ chin and jaw, and used her thumbs to explore his teeth. After 3 sec, she took the grass out of her mouth with her right hand, while maintaining focused grip on Thomas’ mouth with her left hand, and started to meticulously poke the grass in the same dental area as where her thumbs had been. During this time, Noel kept her face within 10 cm of Thomas’ face, never deflecting her gaze from his mouth. After 26 sec of teeth cleaning with the grass, Noel brought the grass to her mouth and inspected the grass orally, seemingly tasting debris removed from Thomas’ mouth. Soon thereafter, Noel engaged in cleaning Thomas’ teeth with the same piece of grass a second time (at least for 5 sec, until the caretakers requested for the recording to end – see Methods), tasting the debris once more (see Figs 1 and 2, and Supplementary Videos 1 and 2).

## Discussion

We consider the cleaning of a corpse’s teeth by Noel noteworthy for several reasons. First and foremost, to date, this behaviour has never been reported in chimpanzees or any other non-human animal species. The records of social tool use in animals are limited to McGrew & Tutin’s^1^,^2^ descriptions of dental cleaning in live chimpanzees. McGrew & Tutin reported how chimpanzees cleaned and even extracted their own and each other’s teeth – an unprecedented behavioural phenomenon in non-human animals that the authors related to the chimpanzees’ experience with object manipulation and their intimate social relationships^2^. The current report extends these observations to dental cleaning of a dead conspecific and corroborates McGrew & Tutin’s perspective on the preconditions of this unusual social behaviour in the form of Noel’s intimate relationship with Thomas. Furthermore, the records of death responses in non-human animals to date do not contain any elements of tool use^8^. In fact, whereas the limited documentations of chimpanzees’ responses in death-related situations range from passive behaviours like resting, to active behaviours like dragging and slapping the dead body^9^, gentle corpse handling in form of tool-assisted dental cleaning (see Supplementary Videos 1 and 2) has never been reported and warrants scientific interest both from an intraspecific and comparative point of view^8^. In particular, the reported corpse handling in chimpanzees may suggest that chimpanzees have evolved in complex social organizations conducive to the emergence of post-mortem affiliation^10^. In other words, chimpanzees may form long-lasting social bonds (e.g. see refs 11 and 12) that continue to influence their behaviour once the bonding partner has died. Like humans, chimpanzees may not treat deceased conspecifics carelessly, but instead handle corpses in a socially meaningful way – i.e. as social beings instead of inanimate objects – especially when this group member is a close associate, as in the reported case^13^. An alternative, non-mutually exclusive explanation for the reported behaviour may be that it emerged from a motivation to learn about death, perhaps fuelled by a curiosity about the unique circumstances^14^. Second, in over 8000 hours of observing the chimpanzees at Chimfunshi, including Noel, we have not observed a single bout of a chimpanzee engaging in tool-assisted teeth cleaning. Notwithstanding the reports by McGrew & Tutin^1^,^2^, this indicates that Noel engaged in a rather unique and meaningful social activity, all the more given that she forfeited abundant high quality food by doing so (i.e. the caretakers tried to lure the chimpanzees away from the body so it could be removed from the enclosure). The fact that Noel preferred to stay close to Thomas over traveling to the high-quality food being offered by the caretakers furthermore casts doubt on the interpretation that Noel cleaned Thomas’ teeth merely to obtain food.

Anecdotal evidence needs to be treated cautiously^15^. However, given the availability of high-quality video footage, we present a valid case of unique non-human animal behaviour which could shed light on the evolution of behaviours that are believed to be typically human. Death responses represent core features of human civilization, with great diversity in mortuary rites found across cultures^16^. In general, for animals critically depending on group living^17^, like humans and chimpanzees, responding to death may be a means to reorganize the social unit, especially when so-called “brokers” die: individuals who play an important role in maintaining group cohesion by connecting sub-groups^18^,^19^. Unfortunately, accounts of dying animals and the responses of their dependents and other group members to their (impending) death are virtually absent from scientific records due to lack of close observation and lack of interest in publishing case studies. The current report on dental corpse cleaning in chimpanzees warrants focused efforts in developing more sophisticated observation techniques to critically capture non-human animals’ (social) behaviour in death-related situations and contributes new behaviour to consider when investigating the evolutionary origins of human death responses.

## Methods

The responses of group members to finding the dead body of Thomas were recorded with two video cameras from the moment that Thomas’ body was discovered until the caretakers requested for the recording to end so that they could make an attempt to climb the fence and retrieve the body from the enclosure. The recording comprised 20 min of uninterrupted filming and was previously used to investigate the group’s responses to the dead body in terms of proximity and individual differences based on association history with the deceased^7^. In the current report, we focused on one peculiar behaviour during the group’s response to Thomas’ dead body – tool use for corpse cleaning – in order to significantly expand the chimpanzee’s ethogram^20^, and highlight how we may miss out on crucial information for reconstructing the evolutionary origins of human mortuary practices by refraining from developing adequate observation techniques to capture non-human animals’ death responses^21^. We inferred from independent interviews with local caregivers (>4 years working with the same chimpanzees) and personal observations by the lead author during a 4-mon period in 2007 that Noel had adopted Thomas by measures of close association, co-feeding (especially during competitive feeding sessions, i.e. the distribution of small portions of high quality food) and co-traveling to nesting sites. All methods were carried out in accordance with the Pan African Sanctuary Alliance guidelines and Chimfunshi Wildlife Orphanage Trust regulations. The research was non-invasive and complied with the ethical guidelines of the Chimfunshi Wildlife Orphanage Trust.

## Additional Information

**How to cite this article:** van Leeuwen, E. J. C. *et al*. Tool use for corpse cleaning in chimpanzees. *Sci. Rep.* **7**, 44091; doi: 10.1038/srep44091 (2017).

**Publisher's note:** Springer Nature remains neutral with regard to jurisdictional claims in published maps and institutional affiliations.

## Supplementary Material

Supplementary Information

Supplementary Video 1

Supplementary Video 2
