# Supplementary material for: Tool use for corpse cleaning in chimpanzees
Source: Sci Rep. 2017 Mar 13;7:44091. doi: 10.1038/srep44091 (PMC5347000; doi:10.1038/srep44091)
Supplement: Supplementary Information [file srep44091-s1.doc]

**Supplementary information (Video legends) for:**

*Title:* Tool use for corpse cleaning in chimpanzees

*Authors:* Edwin J. C. van Leeuwen^1,2*^, Katherine A. Cronin^3^, Daniel B. M. Haun^4^

1: University of St Andrews, School of Psychology & Neuroscience, Westburn Lane, St Andrews, Fife, KY16 9JP, United Kingdom.

2: Max Planck Institute for Psycholinguistics, Wundtlaan 1, 6525 XD Nijmegen, the Netherlands.

3: Lincoln Park Zoo, Lester E. Fisher Center for the Study and Conservation of Apes, IL 60614, Chicago, United States of America

4: University of Leipzig, Department of Early Child Development and Culture and Leipzig Research Center for Early Child Development, Jahnallee 59, Leipzig, 04109, Germany.

**Corresponding author:*

Edwin J. C. van Leeuwen – [ejcvanleeuwen@gmail.com](mailto:ejcvanleeuwen@gmail.com) – +31 621106165

University of St Andrews, School of Psychology & Neuroscience, Westburn Lane, St Andrews, Fife, KY16 9JP, United Kingdom

**Video Legends**

**ESM_1.** **Dental corpse cleaning (entire scene).** When all attending chimpanzees have been lured away from Thomas’ dead body with abundant high-quality food by the local staff, Noel (and her daughter Nina) remains close to Thomas’ body and starts to clean his teeth with a freshly selected piece of grass. Noel and Thomas had a close social bond. Noel associated closely with Thomas' mother TamTam, and when TamTam died, four years before the current observations, Noel adopted Thomas, which was apparent by their close association, co-feeding (especially during competitive feeding sessions, i.e. the distribution of small portions of high-quality food) and co-traveling to nesting sites.

**ESM_2. Dental corpse cleaning (close-up detail).** Noel meticulously pokes with the grass tool between Thomas' teeth.
